# Antioxidant-Enhancing Property of the Polar Fraction of Mangosteen Pericarp Extract and Evaluation of Its Safety in Humans

**DOI:** 10.1155/2016/1293036

**Published:** 2016-09-15

**Authors:** Wichit Suthammarak, Pornpayom Numpraphrut, Ratiya Charoensakdi, Neelobol Neungton, Vachara Tunrungruangtavee, Nattapon Jaisupa, Suwit Charoensak, Primchanien Moongkarndi, Weerasak Muangpaisan

**Affiliations:** ^1^Department of Biochemistry, Faculty of Medicine Siriraj Hospital, Mahidol University, Bangkok, Thailand; ^2^Department of Microbiology, Faculty of Pharmacy, Mahidol University, Bangkok, Thailand; ^3^Department of Psychiatry, Faculty of Medicine Siriraj Hospital, Mahidol University, Bangkok, Thailand; ^4^Department of Preventive and Social Medicine, Faculty of Medicine Siriraj Hospital, Mahidol University, Bangkok, Thailand

## Abstract

Crude extract from the pericarp of the mangosteen (mangosteen extract [ME]) has exhibited several medicinal properties in both animal models and human cell lines. Interestingly, the cytotoxic activities were always observed in nonpolar fraction of the extract whereas the potent antioxidant was often found in polar fraction. Although it has been demonstrated that the polar fraction of ME exhibited the antioxidant activity, the safety of the polar fraction of ME has never been thoroughly investigated in humans. In this study, we investigated the safety of oral administration of the polar fraction of ME in 11 healthy Thai volunteers. During a 24-week period of the study, only minor and tolerable side effects were reported; no serious side effects were documented. Blood chemistry studies also showed no liver damage or kidney dysfunction in all subjects. We also demonstrated antioxidant property of the polar fraction of ME both* in vitro* and* in vivo*. Interestingly, oral administration of the polar fraction of ME enhanced the antioxidant capability of red blood cells and decreased oxidative damage to proteins within red blood cells and whole blood.

## 1. Introduction

The fruit of the tropical evergreen tree* Garcinia mangostana*, or the mangosteen, is regarded as the queen of fruits because of its particularly favorable taste. All parts of the mangosteen, including the seed, pericarp, leaves, and bark, have long been used for medicinal purposes such as treatment of skin infections and diarrhea. Several compounds in the pericarp of the mangosteen have recently been identified, mainly polyphenolic compounds including anthocyanins, xanthones, garcinone E, 8-deoxygartanin, gartanin, prenylated xanthones, and condensed tannin [1, 2]. These compounds have been shown to have many biological activities. In particular, extract from the pericarp of the mangosteen (mangosteen extract [ME]) has been shown to exhibit anti-inflammatory [3–5], anticancer [6–10], antimicrobial [11–14], and antioxidant effects [15–17] both* in vivo* and* in vitro* (for a comprehensive review, see [18]).

Several studies have highlighted the protective effect of ME against oxidative stress. A study by Moongkarndi et al. [15] demonstrated that the water-soluble partition of ME protects the SK-N-SH neuroblastoma cell line from beta-amyloid-induced oxidative stress and alters the proteomic profile of the cells. Sattayasai et al. [19] performed another study that provided compelling evidence for the antioxidant effect of ME. They showed that ME protects SK-N-SH cells from cytotoxicity caused by endogenous hydrogen peroxide and exogenous polychlorinated biphenyl, both of which induce the production of reactive oxygen species (ROS). Intriguingly, supplementation of ME was also shown to prevent memory impairment in scopolamine-treated mice. Moreover, 200 to 800 *µ*g/mL of ME significantly suppressed acetylcholinesterase activity in neurons to approximately 60% of the activity in control cells, which is comparable to the effect of donepezil at its therapeutic level for treatment of patients with Alzheimer's disease.

These findings suggest the potential use of ME as a novel cognitive enhancer in patients with neurodegenerative conditions such as Alzheimer's disease. The use of ME-based food supplements has been gaining increasingly more public attention. However, little is known about whether ME supplementation is indeed safe for humans or benefits human health because most studies of ME supplementation have been conducted using animal models or cell cultures. The cytotoxic activities were always observed in nonpolar fraction of the extract whereas the potent antioxidant was found in polar fraction. In addition, the safety of the polar fraction has never been thoroughly investigated in humans. Several studies reported beneficial effects of the use of *α*-mangostin, the most abundant nonpolar constituent in the pericarp of the mangosteen [20], while some studies showed contradictory results. For instance, dietary *α*-mangostin was shown to exacerbate experimental colitis in a mouse model [21] whereas various studies reported the anticancer and cytotoxic properties of *α*-mangostin [22]. It has also been reported that a single oral dose of *α*-mangostin at 100–1000 mg/kg did not exhibit any harmful effect on the kidney and liver tissues of both male and female ICR mice [23].

Therefore, we conducted the present study to determine the safety of oral administration of the water-soluble partition or polar fraction of ME in human subjects. Healthy Thai volunteers ingested the polar fraction of ME in a capsule preparation daily for 24 weeks. During the study, the volunteers were interviewed regarding ME adherence, any possible side effects, any other medication or supplements, and clinical symptoms. They underwent physical examinations including evaluation of vital signs, body weight, and clinical status. All volunteers also underwent blood testing to evaluate their liver function, kidney function, coagulation status, complete blood count, thyroid function, and plasma electrolyte levels. Electrocardiographic examination and urinary analysis were also performed. The polar fraction of ME in this study was prepared as described previously and possibly contained very low level of *α*-mangostin [19]. The polar fraction of ME was also analyzed by thin-layer chromatography (TLC) for the preparation consistency whereas its antioxidant property was demonstrated by* in vitro *free radical scavenging assay. Finally, we investigated the volunteers' levels of ROS and protein oxidative damage in whole blood and within red blood cells (RBCs).

## 2. Materials and Methods

### 2.1. ME Capsule Preparation

The pericarp of mangosteen was dried at 60°C for 2 days and pulverized by a grinder. It was further extracted by ethanol for 4 days. The extract was evaporated at 45°C by an evaporator. The crude ethanol extract was partitioned with ethyl acetate to 2 : 1 weight : volume for 2 days. The sediment was dissolved with distilled water to 1 : 1 or 1 : 2 volume : volume at 55°C to 60°C for 4 to 6 h. The supernatant was collected by centrifugation at 320 ×g for 20 min and then dried by a spray dryer. The calculated ME powder and cornstarch as an excipient were carefully mixed and placed into hard gelatin capsules (Capsule Products Co. Ltd., Bangkok, Thailand) using a semiautomatic capsule filler at doses of 220 and 280 mg of ME per capsule. The capsules were automatically packed in aluminum foil by a capsule blister-packing machine for moisture protection. At 0, 3, and 6 months after storage, the moisture content of the products was analyzed using a moisture balance (Model MA45; Sartorius, Göttingen, Germany) to ensure control of the capsule products. The physical appearance, weight variation, disintegration, and microbiological contamination were determined according to the standards of the British Pharmacopeia. The biological activities (i.e., antioxidant activities as determined by chemical and cell culture techniques and the fingerprint of ME by thin-layer chromatography and high-performance liquid chromatography) were analyzed to confirm the efficacy of the mangosteen products.

### 2.2. Subject Recruitment

Healthy volunteers were recruited from the Faculty of Medicine, Siriraj Hospital, Mahidol University, Bangkok, Thailand. The interested volunteers were selected according to inclusion and exclusion criteria. The inclusion criteria were (1) age between 20 and 60 years and (2) no previous health problems. The exclusion criteria were (1) those with cardiac, neurological, psychiatric, renal, and liver diseases, cancer, pregnancy or lactation, alcoholic dependence, illicit drug use, and diseases considered by the physician not appropriate to participate in the study and (2) the presence of the following laboratory profiles: absolute neutrophil count < 2 × 10^9^/L, platelet < 100 × 10^9^/L, AST/ALT > 2.5x upper limit, bilirubin > 1.5x upper limit, blood urea nitrogen > 1.5x upper limit, or creatinine clearance < 60 mL/min (Cockcroft and Gault formula). All clinical parameters were obtained at 0, 1, 4, 12, 16, and 24 weeks after beginning the oral ME administration according to standard procedures performed by physicians participating in this study. Participants who developed intolerable side effect or were unwilling to continue in the study or did not comply with the study protocol would be withdrawn from the study. The study was conducted with the understanding and written informed consent of all volunteers. This study was approved by the Siriraj Institutional Review Board (ethical approval number SI333/2012).

### 2.3. ME Capsule Administration and Blood Sample Collection

A 1-month supply of ME capsules or an adequate number of capsules to last until the next visit was given to each volunteer. During the first 3 months, volunteers who weighed <55 kg took a single 220 mg ME capsule daily, while those who weighed >55 kg took a single 280 mg ME capsule daily. Dosing was calculated based on the previous study on mice from Sattayasai et al. [19]. The dose in mice was converted to the human equivalent dose according to “the guidance for industry: estimating the maximum safe starting dose in initial clinical trials for therapeutics in adult healthy volunteers” from US Department of Health and Human Services Food and Drug Administration Center for Drug Evaluation and Research, CDER, July 2005. After 3 months, all of the volunteers received a double dose daily. All volunteers underwent collection of 4 mL blood samples at 0, 1, 4, 12, 16, and 24 weeks after beginning the oral ME administration.

### 2.4. Separation of ME by TLC and Screening of Antioxidant Activity

The slightly modified TLC was applied [24]. The extract was dissolved in methanol and then subjected onto a 20 × 20 cm normal phase aluminum plate (silica gel 60 F254, Merck; St. Louis, MO). The plate was developed in the mobile phase consisting of ethyl acetate : 70% ethanol in a ratio of 10 : 1 volume : volume. The isolated bands were visualized under UV light. To screen the antioxidant activity of each isolated band of ME, stable free radical, 2,2-diphenyl-1-picrylhydrazyl radical (DPPH), was applied. The developed plate was sprayed by 0.3 mM DPPH in methanol. The formation of yellow color indicated the antioxidant activity on the basis of the formation of reduced DPPH.

### 2.5. *In Vitro* Assay for Antioxidant Property of ME

The assay was performed as described by Moongkarndi et al. [25]. In brief, isolated band A from TLC separation was dissolved in methanol to various concentrations (0, 2.5, 5, 10, 20, 50, and 100 *μ*g/mL). To 100 *μ*L of extract solution, 100 *μ*L of fresh 0.4 mM DPPH solution in methanol was added. The experiment was conducted in a 96-well plate (*n* = 3 independent samples). The reaction was incubated in darkness at room temperature for 30 min. The absorbance was measured at 517 nm by microplate reader (Anthos 2010, Anthos Labtec Instruments GmbH). Vitamin C was used as positive control. The degree of discoloration indicates the scavenging potential of the antioxidant property. The scavenging activity was calculated by [1 − (*A*_1_ − blank)/*A*_0_ − blank] × 100, where *A*_1_ was the absorbance of DPPH in the presence of the extracts, *A*_0_ represented the absorbance of DPPH solution without extract addition, and blank was the absorbance without DPPH solution.

### 2.6. Measurement of ROS in RBCs

The heparinized blood was diluted with Ca^2+^- and Mg^2+^-free phosphate buffered saline (PBS) (Sigma, St. Louis, MO, USA) to a concentration of 1 × 10^6^ RBCs/mL. The RBCs were incubated with 0.4 mM of 2′-7′-dichlorofluorescein diacetate (DCFH-DA) (Sigma) dissolved in methanol (Fluka; Sigma-Aldrich, St. Louis, MO, USA). After incubation at 37°C for 15 min in a humidified atmosphere of 5% carbon dioxide in air, the cells were washed and resuspended in PBS to the original cell concentration. The RBC suspension was analyzed by incubation with or without 2 mM of freshly prepared hydrogen peroxide. After incubation at room temperature for 20 min, the samples were analyzed by a fluorescence-activated cell sorter (FACSCalibur; Becton-Dickinson, Immunofluorometry Systems, Mountain View, CA, USA). The RBCs were passed at a rate of about 1,000 cells/s. A 488 nm argon laser beam was used for excitation [26]. The absolute fluorescence intensity of the 2′,7′-dichlorofluorescein (DCF), which represented the ROS level in the RBCs, was interpreted on a two-parameter dot plot of the side light scatter and forward light scatter of the population. Cell fluorescence was measured using logarithmic amplification. Green fluorescence of 10,000 cells was then measured using linear amplification. The arithmetic mean fluorescence channel was derived by CellQuest® software (Becton-Dickinson).

### 2.7. Determination of Protein Oxidative Damage by Anti-4-Hydroxynonenal Antibody Staining

To prepare RBCs foranti-4-hydroxynonenal (anti-HNE) antibody staining, whole blood was mixed with an equal volume of PBS and then centrifuged at 800 ×g for 5 min at 4°C. The pellet containing the RBCs was collected and washed three times with PBS. Finally, distilled water was added to suspend and lyse the cells, and the solution was stored at −80°C until use.

Either whole blood or RBCs were denatured in sample buffer (100 mM Tris-HCl, pH 6.8, 10% glycerol, 4% SDS, 0.002% bromophenol blue, 100 mM dithiothreitol, and 5% 2-mercaptoethanol) and then boiled at 95°C for 5 min. The samples were dotted manually in duplicate (one for HNE and one for *β*-actin detection) onto nitrocellulose membrane with amount of total protein 0.5 *μ*g per dot based on Bradford assay and left to dry in air. The nitrocellulose membranes were then blocked in 5% nonfat milk in PBS with 0.01% Tween-20 (PBST) with 0.01% NaN_3_ at 4°C for 1 h and subsequently incubated with either primary antibody: anti-HNE antibody (Millipore, Billerica, MA, USA) or anti-actin-3 antibody (Santa Cruz Biotechnology, Santa Cruz, CA, USA) for 2 h. Then, the membranes were washed 3 times with PBST and subsequently incubated with horseradish peroxidase-conjugated secondary antibodies (Santa Cruz Biotechnology, Santa Cruz, CA, USA) in the same solution without NaN_3_ at room temperature for 2 h. After incubation with secondary antibody, the membranes were washed with PBST for 3 times. A chemiluminescence substrate (SuperSignal West Pico; Thermo Fisher Scientific, Inc., Rockford, IL, USA) was used to develop the reactions. The fluorescence generated from the reaction was visualized by exposing to the film. The band intensities of HNE and actin-3 from the samples were quantitated by ImageJ software (US National Institutes of Health, Bethesda, MD, USA).

The negative control dots were also performed without primary antibody for both whole blood and RBCs samples; no signal was detected.

### 2.8. Data Analysis

Analysis of variance was performed to analyze groups of data for significant differences. Unpaired Student's *t*-test was then used to calculate statistical significance of specific pairs if a difference was demonstrated with analysis of variance.

## 3. Results

### 3.1. Biosafety Assessment and Side Effects of Oral Administration of ME

The safety of oral administration of the polar fraction of ME was assessed in 11 healthy human subjects. The demographic data of the subjects are shown in Table 1. The plasma electrolyte levels, lipid profiles, liver function enzyme levels, thyroid hormone levels, coagulograms, and complete blood counts were measured from whole blood samples of all subjects at 0, 1, 4, 12, 16, and 24 weeks after beginning oral administration of the polar fraction of ME. The results showed no significant alterations in these parameters (Table 2). Additionally, the side effects of oral administration of the polar fraction of ME were determined throughout the study. All subjects reported minor and tolerable side effects as shown in Table 3.

### 3.2. Separation of ME by TLC and Screening of Antioxidant Activity

**Figure 1:**
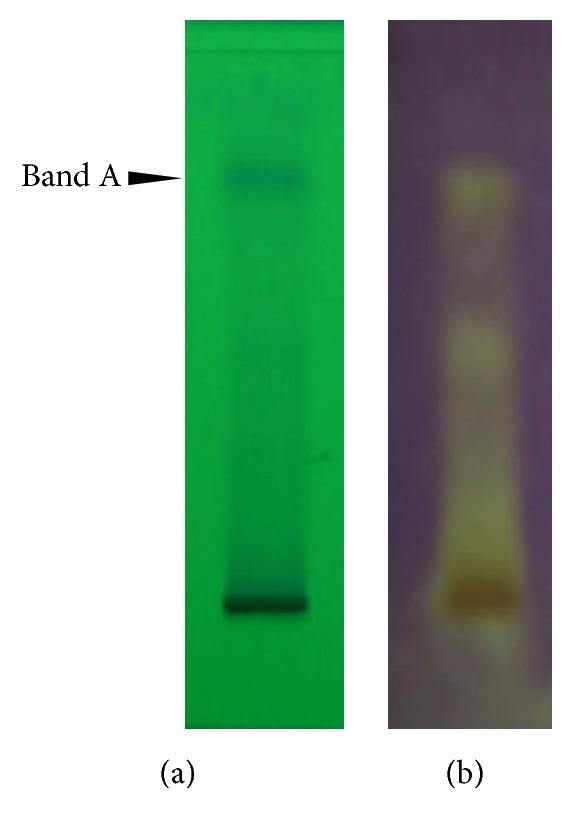
Separation of ME by TLC and antioxidant properties of its constituents. (a) ME was separated by TLC and visualized under UV at 254 nm. Major compounds appeared in a single band at *R*_*f*_ = 0.75 (band A). (b) Screening of antioxidant activity was done by applying DPPH onto the TLC plate. Scavenging of DPPH was shown in yellow staining indicating antioxidative activity of ME.

TLC chromatogram of the polar fraction of ME visualized under UV light at 254 nm revealed a remarkable band that had *R*_*f*_ value of approximately 0.75 (band A). After spraying with 0.3 mM DPPH solution, several yellow spots, which indicated antioxidant properties, were observed (Figure 1).

### 3.3. *In Vitro* Antioxidant Assay of ME

**Figure 2:**
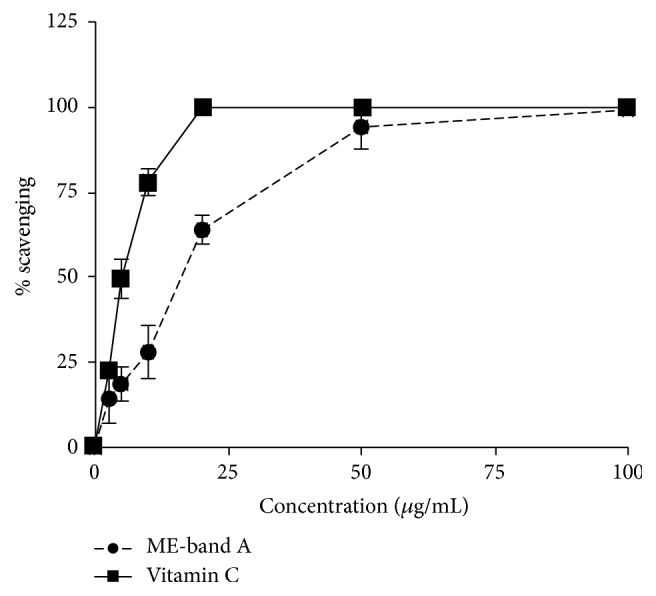
Quantitative analysis of antioxidant activity of ME. DPPH assay was performed on the extract of band A (ME-band A) from TLC. Vitamin C was used as a positive control for comparison. Antioxidant activities (% scavenging) of both ME and vitamin C, calculated as described in Materials and Methods, were dose-dependent. The IC_50_ values of ME and vitamin C were 16.03 ± 1.44 *μ*g/mL and 5.16 ± 0.92 *μ*g/mL, respectively. Error bars represent SEM.

Quantitative analysis of antioxidant activity of the polar fraction of ME was performed on the extract of band A by DPPH assay (Figure 2). Antioxidant activity was shown in dose-dependent manner. The IC_50_ values of band A extract and vitamin C (positive control) were 16.03 ± 1.44 *μ*g/mL and 5.16 ± 0.92 *μ*g/mL, respectively.

### 3.4. Antioxidant Capacity of RBCs

**Figure 3:**
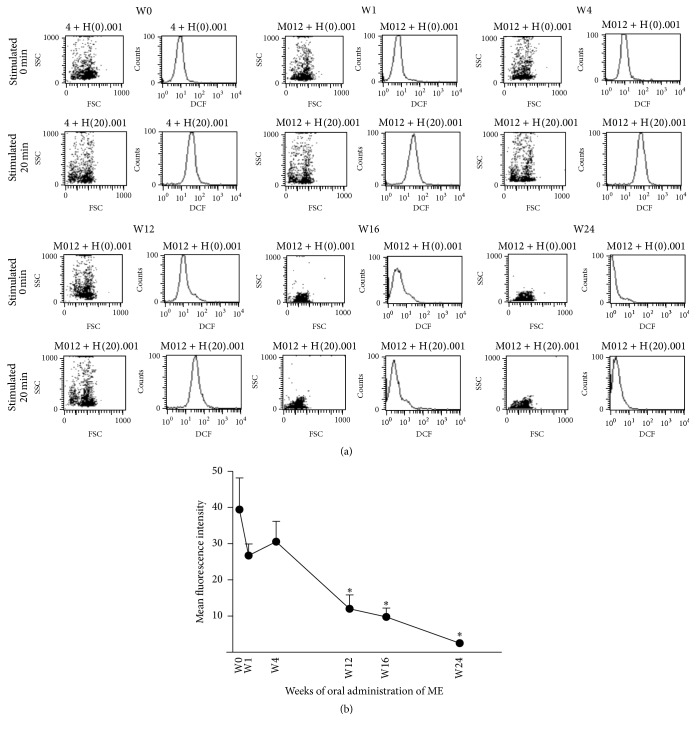
Effect of oral administration of ME on antioxidant capacity of RBCs. RBCs were isolated from the peripheral blood of the subjects at 0, 1, 4, 12, 16, and 24 weeks after beginning oral administration of ME. Hydrogen peroxide was an exogenous ROS used to stimulate the response of the RBC antioxidant system. (a) A representative measurement of the level of ROS within the RBCs which was determined by the mean fluorescent signal of DCF emitted from hydrogen peroxide-incubated RBCs. (b) Quantitative analysis of the mean fluorescent signal of DCF from all samples during the entire period of the study. Data are presented as means (*n* = 11). Error bars represent SEM. The asterisk indicates statistical significance: *p* < 0.05 in comparison with week 0 (*t*-test).

The antioxidant capacity of RBCs was determined by measuring the fluorescent signal of DCF from the RBCs after incubation with hydrogen peroxide, which was used as an exogenous ROS. The RBCs from the subjects taking the polar fraction of ME exhibited a significant decrease in the mean fluorescent intensity of DCF starting in week 12 of oral administration of ME (Figure 3). This suggests that ME increases the antioxidant capacity of RBCs. This effect was further accentuated through the end of the study.

### 3.5. Oxidative Damage to Proteins in RBCs and Whole Blood

**Figure 4:**
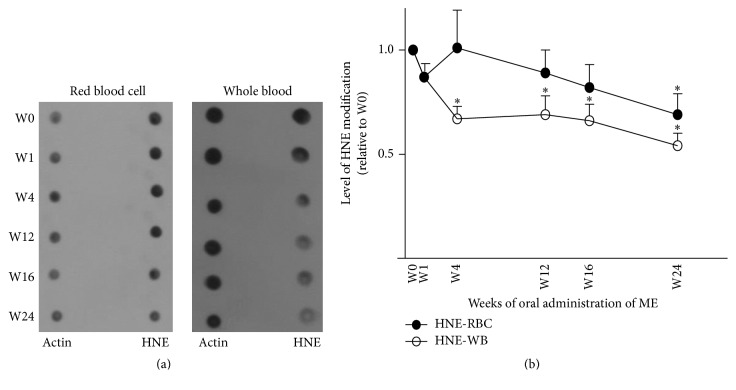
Oral administration of ME resulted in decreased protein oxidative damage in RBCs and whole blood. The RBCs and whole blood of the subjects were collected at 0, 1, 4, 12, 16, and 24 weeks after beginning oral administration of ME. (a) A representative blot shows HNE staining using a western blotting technique to determine the protein oxidative damage in RBCs and whole blood. Actin was used as a loading control. (b) Quantitative analysis of the extent of HNE modification to RBCs (HNE-RBC) and whole blood (HNE-WB) after oral administration of ME was carried out as that relative to week 0. Data are presented as means (*n* = 11). Error bars represent SEM. The asterisk indicates statistical significance: *p* < 0.05 in comparison with week 0 (*t*-test).

Oral administration of ME was associated with a significant decrease in oxidative damage to proteins in both RBCs and whole blood. Quantification of oxidative damage to these proteins was achieved by normalizing HNE staining to actin. As shown in Figure 4, a significant decrease in protein oxidative damage appeared in weeks 4 and 24 for whole blood and RBCs, respectively.

## 4. Discussion

Several compounds in the extract from mangosteen pericarp have recently been identified and shown to have beneficial effects on human health. However, the cytotoxic activities were always observed in nonpolar fraction of the extract, that is, *α*-mangostin, whereas the potent antioxidant was found in polar fraction. In addition, the safety of the polar fraction has never been thoroughly investigated in humans. In this study, we investigated the safety of oral administration of the polar fraction of ME in Thai subjects. For the entire 24 weeks of the study, only minor side effects were reported. These can be categorized into gastrointestinal, skin, respiratory, and general side effects. The most frequently reported side effects were related to the gastrointestinal system. We suspected that such side effects might be associated with an antibacterial property of the compounds from the mangosteen pericarp, which may have led to the perturbation of gut microbes. Nonetheless, these side effects accounted for only a few episodes and were reportedly tolerable by all subjects.

The present study also showed that oral administration of the polar fraction of ME poses no threat to vital organs including the liver, kidney, and thyroid gland. The subjects' liver enzyme levels, lipid profiles, coagulogram parameters, blood urea nitrogen level, creatinine level, urine protein level, plasma electrolyte levels, and thyroid hormone levels remained normal for the entire 24 weeks of the study. Additionally, all subjects' complete blood counts and blood pressures remained unchanged. Although two subjects showed slightly increased liver enzymes in the fourth week of the study, the enzyme levels spontaneously returned to the normal range thereafter. Notably, a study by Stern et al. [27] revealed that obese subjects with a body mass index of 30 to 40 kg/m^2^ exhibited a significant net reduction in body weight (3.74 kg; *p* < 0.0001) after taking capsules containing* Sphaeranthus indicus* and* G. mangostana* for 8 weeks compared with subjects taking placebo. The discrepancy in the subjects' body weights between that study and the present study might derive from the volunteers in our study having a normal BMI or from the weight-loss property of* S. indicus*.

We performed TLC on ME in order to test for preparation consistency and to demonstrate its essential compounds. It was shown that ME was composed of at least one group of major compounds, which consistently appeared as a single band on the chromatogram at *R*_*f*_ = 0.75. Such a high *R*_*f*_ suggested highly organic-volatile property. We are currently identifying the chemical compounds in this band (data not shown). Further* in vitro* analysis of these major compounds revealed that they exhibited approximately one-third of antioxidant activity of vitamin C. Therefore, ME is likely to possess considerably high antioxidant activity.

Antioxidant property of the polar fraction of ME observed in our* in vitro* experiments was shown to exert biological impact. The 24-week period of oral administration of the polar fraction of ME in the present study resulted in a significant increase in the antioxidant capacity of RBCs, which was determined by the fluorescent signal from DCF emitted from hydrogen peroxide-incubated RBCs. A significant increase in the antioxidant capacity (i.e., decreased DCF signal) of RBCs could be detected from week 12 of oral administration of the polar fraction of ME. Therefore, our results suggest for the first time that extract from mangosteen pericarp can augment the antioxidant system within human RBCs, probably due to its ability to neutralize ROS.

We further investigated whether and how such an increased antioxidant capacity affects protein oxidative damage in RBCs and whole blood. Protein oxidative damage was determined by the degree of HNE modification. HNE is a byproduct of lipid peroxidation, which is an indicator of oxidative stress. HNE can react with lysine, histidine, or cysteine residue in proteins to form adducts, resulting in structural and functional changes to the proteins [28, 29]. HNE is also reportedly involved in the pathophysiology of many diseases [30–32]. We speculated that if RBCs have an improved antioxidant capacity, lipid peroxidation should be reduced and HNE modification to proteins should thus decrease. Consistent with our speculation, oral administration of the polar fraction of ME led to a significant decrease in HNE modification in whole blood and RBCs starting in weeks 4 and 24, respectively. Notably, the significant decrease in HNE modification of whole blood proteins appeared earlier than that of RBC proteins. This may be explained by the faster turnover rate of albumin [33], the most abundant protein in serum, in comparison to the lifespan of RBCs [34]. This result provides further supporting evidence in human subjects of the antioxidant property of extract from mangosteen pericarp.

## 5. Conclusions

We have demonstrated for the first time in human subjects that the polar fraction of extract from mangosteen pericarp is adequately safe for oral administration for up to 24 weeks. Major components of the polar fraction of extract from mangosteen pericarp exhibited considerably high antioxidant activity according to the* in vitro* antioxidant assay. Additionally, the polar extract from mangosteen pericarp appears to significantly increase the antioxidant capacity of RBCs and significantly decrease protein oxidative damage in RBCs and whole blood. It might alleviate oxidative damage in other tissues as well. Our results suggest that the antioxidant capacity of the polar fraction of mangosteen pericarp extract could come from its ability to neutralize ROS. However, whether some compounds in this extract can also upregulate antioxidant enzyme is still unknown. It would be interesting to further quantitate the expression level of antioxidant enzymes. In all, the polar fraction of extract from mangosteen pericarp might benefit human patients with pathological conditions related to oxidative stress.

## Figures and Tables

**Table 1:** Demographic data of the subjects.

Demographic data	*n*	%
*Sex*		
Male	5	45.5
Female	6	54.5

*Age in years (* $\overset{-}{x}$ * = 32)*		
20–30	4	36.4
31–40	6	54.5
>40	1	9.1

*Occupation*		
Employee	2	18.2
Government officer	1	9.1
University worker	6	54.5
Others	2	18.2

*Alcohol consumption*		
Stopped drinking for >3 mo	2	18.2
Drinking less than once/mo	7	63.6
Never	2	18.2

*Smoking*		
Never	11	100.0

*Herb/vitamin supplement*		
Consuming	1	9.1
Never	10	90.9

**Table 2:** Subject data and biochemical parameters for assessment of safety of oral administration of ME. Blood and urine samples were collected from the subjects in 0, 1, 4, 12, 16, and 24 weeks after beginning oral administration of ME. The biochemical parameters were measured as tabulated. Body mass index and blood pressure were also determined. All parameters were collected and calculated from 11 healthy individuals.

Test	Subject data and laboratory safety assessment, mean (SEM)					
	Day 0	1 week	4 weeks	12 weeks	16 weeks	24 weeks
Glucose (mg/dL)	86.73 (1.64)	85.00 (1.67)	83.09 (1.45)	81.45 (3.04)	80.91 (1.46)	85.00 (2.53)
Cholesterol (mg/dL)	206.54 (13.10)	196.00 (12.95)	201.09 (14.32)	193.45 (10.34)	190.63 (13.61)	192.45 (24.74)
Triglycerides (mg/dL)	78.00 (11.15)	77.18 (19.58)	81,27 (20.11)	87.18 (17.27)	103.54 (30.80)	92.27 (22.65)
HDL-CHOL (mg/dL)	62.90 (2.94)	60.45 (2.52)	59.45 (2.13)	59.72 (2.08)	57.81 (2.26)	59.45 (1.95)
LDL-calculated (mg/dL)	128.03 (12.00)	120.10 (10.62)	125.38 (12.34)	116.29 (8.95)	112.10 (9.13)	114.54 (10.80)
BUN (mg/dL)	12.84 (1.63)	11.26 (0.58)	12.43 (1.03)	11.82 (0.82)	12.23 (0.88)	12.17 (1.18)
Creatinine (mg/dL)	0.86 (0.06)	0.84 (0.04)	0.81 (0.05)	0.82 (0.04)	0.82 (0.04)	0.81 (0.04)
Sodium (mmol/L)	141.27 (0.57)	141.54 (0.89)	141.36 (0.51)	140.09 (0.47)	141.09 (0.41)	140.18 (0.38)
Potassium (mmol/L)	3.75 (0.06)	3.72 (0.09)	3.75 (0.07)	3.58 (0.08)	3.56 (0.06)	3.63 (0.08)
Chloride (mmol/L)	102.79 (0.45)	103.08 (0.56)	102.50 (0.50)	101.79 (0.71)	103.23 (0.55)	102.93 (0.36)
Bicarbonate (mmol/L)	28.14 (0.85)	24.92 (0.70)	26.50 (1.04)	25.43 (0.70)	24.85 (1.01)	24.14 (0.71)
Total bilirubin (mg/dL)	0.63 (0.14)	0.60 (0.11)	0.49 (0.08)	0.59 (0.11)	0.60 (0.14)	0.57 (0.10)
Direct bilirubin (mg/dL)	0.21 (0.04)	0.23 (0.03)	0.20 (0.02)	0.24 (0.04)	0.24 (0.04)	0.22 (0.03)
AST (SGOT) (U/L)	20.81 (1.89)	19.45 (2.07)	30.54 (8.04)	18.00 (1.80)	19.09 (1.46)	17.63 (2.83)
ALT (SGPT) (U/L)	19.18 (4.12)	17.00 (3.68)	31.27 (10.40)	18.45 (4.54)	15.63 (2.61)	20.45 (7.71)
ALP (U/L)	60.72 (2.68)	59.27 (3.14)	63.63 (3.17)	62.45 (2.05)	64.09 (3.74)	62.54 (3.14)
T3 (ng/dL)	99.42 (3.27)	104.62 (3.53)	97.45 (3.16)	97.35 (3.82)	99.26 (6.33)	92.62 (3.72)
TSH (*μ*IU/mL)	2.15 (0.32)	2.01 (0.32)	2.09 (0.42)	2.03 (0.36)	2.07 (0.28)	2.16 (0.30)
FT4 (ng/dL)	1.32 (0.05)	1.28 (0.05)	1.26 (0.05)	1.22 (0.02)	1.18 (0.03)	1.22 (0.04)
Hemoglobin (g/dL)	13.60 (0.31)	13.25 (0.39)	13.45 (0.31)	13.55 (0.37)	13.50 (0.40)	13.50 (0.43)
Hematocrit (%)	42.19 (1.00)	40.91 (1.04)	41.32 (1.05)	41.43 (1.10)	40.51 (1.25)	40.64 (1.21)
WBC count (×10^3^/*μ*L)	5.97 (0.36)	5.77 (0.33)	5.94 (0.26)	6.44 (0.32)	5.70 (0.37)	5.43 (0.29)
Platelet count (×10^3^/*μ*L)	246.27 (13.14)	240.45 (14.30)	255.36 (14.13)	243.54 (12.65)	238.27 (12.20)	239.72 (10.64)
Absolute neutrophils (×10^3^/*μ*L)	3.09 (0.23)	3.19 (0.26)	3.27 (0.14)	3.88 (0.44)	3.12 (0.28)	2.84 (0.20)
PT (sec)	12.14 (0.21)	12.08 (0.21)	11.99 (0.24)	12.23 (0.19)	12.48 (0.27)	12.62 (0.22)
aPTT (sec)	28.53 (0.60)	28.19 (0.76)	28.61 (0.69)	28.97 (0.61)	29.20 (0.81)	29.70 (0.74)
Urine protein (Neg–trace)	54.5%	72.7%	72.7%	81.8%	72.7%	54.5%
Body mass index	21.74 (1.12)	21.86 (1.12)	21.64 (1.07)	22.07 (1.14)	21.91 (1.12)	21.89 (1.13)
Arterial blood pressure (mm Hg)	117/72	117/76	117/77	119/73	118/72	113/73

HDL-CHOL, high-density lipoprotein cholesterol; LDL, low-density lipoprotein; BUN, blood urea nitrogen; AST (SGOT), aspartate aminotransferase (serum glutamic oxaloacetic transaminase); ALT (SGPT), alanine aminotransferase (serum glutamic pyruvic transaminase); ALP, alkaline phosphatase; T3, triiodothyronine; TSH, thyroid-stimulating hormone; FT4, free thyroxine; WBC, white blood cell; PT, prothrombin time; aPTT, activated partial thromboplastin time.

**Table 3:** Side effects of oral administration of ME. The subjects were asked to report the side effects after oral administration of ME at each visit. The number of episodes of each side effect prior to and after double doses was collected and calculated as the percentage of the total episodes of 110 (11 participants × 10 visits).

Symptom	Before (single dose)		After (double doses)	
	Number of episodes	%	Number of episodes	%
Tiredness/sleepiness	4	3.6	0	0.0
Constipation	4	3.6	1	0.9
Dry throat	2	1.8	3	2.7
Headache	1	0.9	4	3.6
Flu-like symptoms	2	1.8	0	0.0
Abnormal stool	4	3.6	0	0.0
Abdominal discomfort	3	2.7	0	0.0
Cough	2	1.8	0	0.0
Weight loss	2	1.8	2	1.8
Nasal congestion	2	1.8	0	0.0
Abdominal bloating	2	1.8	1	0.9
Dizziness	1	0.9	0	0.0
Salivation	1	0.9	0	0.0
Nausea/vomiting	1	0.9	1	0.9
Diarrhea	1	0.9	1	0.9
Skin rash	1	0.9	0	0.0
Abnormal urination	1	0.9	0	0.0
Malaise	1	0.9	0	0.0
